# Double-Stranded RNA High-Throughput Sequencing Reveals a New Cytorhabdovirus in a Bean Golden Mosaic Virus-Resistant Common Bean Transgenic Line

**DOI:** 10.3390/v11010090

**Published:** 2019-01-21

**Authors:** Dione M. T. Alves-Freitas, Bruna Pinheiro-Lima, Josias C. Faria, Cristiano Lacorte, Simone G. Ribeiro, Fernando L. Melo

**Affiliations:** 1 Embrapa Recursos Genéticos e Biotecnologia, Brasília 70770-917, Brazil; dionebio@gmail.com (D.M.T.A.-F.); pinheiro.limab@gmail.com (B.P.-L.); cristiano.lacorte@embrapa.br (C.L.); 2 Departamento de Biologia Celular, Universidade de Brasília, Brasília 70910-900, Brazil; 3 Embrapa Arroz e Feijão, Goiânia 75375-000, Brazil; josias.faria@embrapa.br; 4 Departamento de Fitopatologia, Universidade de Brasília, Brasília 70910-900, Brazil

**Keywords:** common bean, *Phaseolus vulgaris*, rhabdovirus, cytorhabdovirus, dsRNA high throughput sequencing

## Abstract

Using double-strand RNA (dsRNA) high-throughput sequencing, we identified five RNA viruses in a bean golden mosaic virus (BGMV)-resistant common bean transgenic line with symptoms of viral infection. Four of the identified viruses had already been described as infecting common bean (cowpea mild mottle virus, bean rugose mosaic virus, Phaseolus vulgaris alphaendornavirus 1, and Phaseolus vulgaris alphaendornavirus 2) and one is a putative new plant rhabdovirus (genus *Cytorhabdovirus*), tentatively named bean-associated cytorhabdovirus (BaCV). The BaCV genome presented all five open reading frames (ORFs) found in most rhabdoviruses: nucleoprotein (N) (ORF1) (451 amino acids, aa), phosphoprotein (P) (ORF2) (445 aa), matrix (M) (ORF4) (287 aa), glycoprotein (G) (ORF5) (520 aa), and an RNA-dependent RNA polymerase (L) (ORF6) (114 aa), as well as a putative movement protein (P3) (ORF3) (189 aa) and the hypothetical small protein P4. The predicted BaCV proteins were compared to homologous proteins from the closest cytorhabdoviruses, and a low level of sequence identity (15–39%) was observed. The phylogenetic analysis shows that BaCV clustered with yerba mate chlorosis-associated virus (YmCaV) and rice stripe mosaic virus (RSMV). Overall, our results provide strong evidence that BaCV is indeed a new virus species in the genus *Cytorhabdovirus* (family *Rhabdoviridae*), the first rhabdovirus to be identified infecting common bean.

## 1. Introduction

Common bean (*Phaseolus vulgaris* L.) is an economically important leguminous crop grown worldwide. It is a staple food for human consumption and the most consumed legume worldwide. Brazil is the third largest producer and one of the major consumers of common beans in the world [1]. In Brazil, common bean is cultivated in farms with large areas using high technology as well as by small farm holders and as a subsistence crop by a large number of families. Therefore, it has both economic and social impact as a cash crop and as a source of protein and other nutrients for families relying on subsistence farming. Several factors, such as susceptibility to diseases and insect pests, may impact bean production and consequently reduce food security and farmer income [2].

Different viruses have been reported infecting common beans. Some of them are asymptomatic, such as the double-strand RNA (dsRNA) virus Phaseolus vulgaris alphaendornavirus 1 (PvEV-1; family *Endornaviridae*, genus *Alphaendornavirus*), a seed-borne virus [3]. Other viruses are quite severe, causing significant yield losses and reducing the productivity and crop quality. These include bean golden mosaic virus and bean golden yellow mosaic virus (BGMV and BGYMV, respectively; family *Geminiviridae*, genus *Begomovirus*) [4,5], bean common mosaic virus (BCMV; family *Potyviridae*, genus *Potyvirus*) [6], and cowpea mild mottle virus (CPMMV; family *Betaflexiviridae,* genus *Carlavirus*) [7].

Among bean-infecting viruses, BGMV is the primary limiting factor to bean production in Brazil and causes up to 100% crop losses [2,8]. To overcome the limitations imposed by the golden mosaic disease, a transgenic bean line has been generated with resistance to BGMV. The transgenic BGMV-resistant line developed by Bonfim et al. [9] relies on the RNA silencing mechanism to contain virus replication and accumulation in the plants. Under field conditions, homozygous transgenic plants displayed 100% immunity to BGMV infection [10].

Plant infection by multiple viruses is common in nature and is frequently reported [11,12]. During multiple infections, viruses can interact among themselves and depending on the virus, host plant, and temporal and spatial factors, the disease phenotype will reflect the type of interaction, which may range from synergism to antagonism [11,12,13].

It was already known that BGMV-resistant transgenic bean lines are susceptible to CPMMV [2]. However, the lack of the golden mosaic symptoms in the transgenic lines allowed the detection of symptoms of mild chlorosis, vein banding, and mottling, indicating that CPMMV is present in high incidences of up to 69% in these plants under field conditions [2].

More recently, transgenic lines in field experiments presented severe symptoms of mosaic, including leaf distortion, crinkling, and vein necrotic enations. These more severe symptoms could be the outcome of multiple virus infection in these plants. Diagnostic PCR tests were negative for the begomoviruses reported as infecting beans in Brazil (BGMV, Macroptilium yellow spot virus (MaYSV), and Sida micrantha mosaic virus (SiMMV)). Therefore, to test for the presence of viruses with RNA genomes in the BGMV-resistant common bean transgenic line CNFCT16207, we performed high-throughput sequencing (HTS) of enriched dsRNA samples. We were able to identify five viruses, including four known viruses from distinct genera (*Alphaendornavirus, Carlavirus*, and *Comovirus*) and one newly discovered virus in the genus *Cytorhabdovirus* (family *Rhabdoviridae*), herein named bean-associated cytorhabdovirus (BaCV).

## 2. Materials and Methods

### 2.1. Plant Material

Leaves from four transgenic BGMV-resistant common bean breeding line CNFCT16207 plants showing severe symptoms of crinkling were collected from experimental fields at Embrapa Arroz e Feijão, located in Santo Antonio de Goiás, Goiás State, Brazil, in 2014. The leaves were separated into two samples and stored in a −80 °C freezer. A fraction of the fresh leaves was ground in inoculation buffer (0.1 M phosphate buffer with 0.1 % sodium sulfite) and the extract was used for the mechanical inoculation of four common bean cv. Jalo Precoce and four transgenic BGMV-resistant line CNFCT16207 seedlings. Plants were kept in a greenhouse with temperatures varying between 24 °C and 36 °C. The inoculated plants were observed for up to 50 days and regularly checked for symptom development. Leaves were collected for dsRNA extraction.

### 2.2. dsRNA Extraction

dsRNA extraction was done based on modified protocols of Valverde et al. [14] and Castillo et al. [15]. Frozen leaves from field samples and fresh leaves from inoculated bean plants (cv. Jalo Precoce and CNFCT16207 line) were used for dsRNA preparation. Two grams of foliar tissue was frozen in liquid nitrogen and pulverized in a mortar and pestle. Total nucleic acids were extracted with 1× STE buffer (0.1 M NaCl, 0.05 M Tris-base, 0.001 M EDTA, pH8), 1% of SDS, 1% of β-mercaptoethanol, and phenol:chloroform:isoamyl alcohol (25:24:1). The aqueous phase volume was measured and absolute ethanol was added to a final concentration of 16%. The resulting mixture was loaded in a column containing one gram of cellulose (C6288-Sigma-Aldrich, St. Louis, MO, USA) equilibrated with wash buffer (0.1 M NaCl, 0.05 M Tris-base, 0.001 M EDTA, pH 8, 16% ethanol). The mixture was passed through the cellulose column twice. The column was washed twice with wash buffer. dsRNA was eluted with STE 1× and precipitated with 0.1 volume of 3 M sodium acetate and 2.5 volumes of ethanol at −20 °C overnight. The precipitate was dissolved in 100 µL of RNase free H_2_O and stored at −20 °C. The presence of dsRNA was determined by agarose gel electrophoresis.

### 2.3. High-Throughput Sequencing and Assembly

dsRNA from all samples (*n* = 12) was pooled in a single tube (200 ng in total) and sequenced using the MiSeq sequencing platform (Illumina, San Diego, CA, USA) at the Universidade Católica de Brasília (UCB). Total dsRNA was converted to cDNA using random hexamers, fragmented into libraries using the transposon-based Nextera™ DNA Sample Prep Kit and sequenced using MiSeq Reagent Kits v2 (2 × 150 bp) (Illumina, San Diego, CA, USA). The raw reads were quality trimmed and assembled de novo using CLC Genomics Workbench version 6.3. The resulting contigs were compared to the complete viral RefSeq database (release 89, July 9, 2018) using Blastx [16] implemented in Geneious version 9.1.2 [17]. All sequences with hits matching the viral database were then subjected to a Blastx search against the nr database to confirm true viral contigs and exclude false positives (e.g., as endogenous viral elements and cellular RNA-dependent RNA polymerases). To confirm the assembly results and further extend incomplete genomes, trimmed reads were mapped back to the viral contigs and reassembled, until genome completion or no further extension. The final sequences of the virus genomes were obtained from the majority consensus of the mapping assembly and annotated using Geneious version 9.1.2 [17].

### 2.4. Viral-Specific RT-PCR, Cloning, and Sequencing

Based on the assembled contigs, specific primers were designed for each virus identified (Table 1). Each sample was submitted to a virus-specific RT-PCR using the SuperScript^®^ III One-Step RT-PCR kit (Life Technologies, Carlsbad, CA, USA). The reaction mixes consisted of 1 to 2.5 µL of dsRNA, 6.3 µL of 2× reaction mix, 1 µL of each forward and reverse primers (10 μM), and 0.4 µL of SuperScript^®^ III RT/Platinum^®^ Taq mix in a final volume of 12.5 µL. The cycling conditions were 1 cycle for cDNA synthesis at 53 °C for 20 min; 1 cycle for denaturation at 94 °C for 2 min 30 s; 35 cycles for PCR amplification at 94 °C for 20 s, specific primer Tm °C (Table 1) for 35 s, and 68 °C for 1 min per kilobase; and a final extension step at 68 °C for 10 min. The presence of virus-derived PCR products was verified by gel electrophoresis. Single RT-PCR product of each specific virus genome was gel-purified and cloned into pCR™2.1-TOPO^®^ vector (Life Technologies, Carlsbad, CA, USA). Four clones for each virus-derived fragment were Sanger-sequenced at Macrogen Inc. (Seoul, Korea).

### 2.5. 5′/3′ Rapid Amplification of cDNA Ends (RACE)

To determine the 5′- and 3′-terminal sequences of BaCV genome, rapid amplification of cDNA ends (RACE) was performed according to the method described previously [18,19]. The 3′ end was confirmed by the addition of a poly-A tail to the total RNA using the *Escherichia coli* poly(A) polymerase (New England Biolabs, Ipswich, MA, USA); then, the cDNA was synthesized using an oligo(dT) primer with an anchor sequence, M10PacIT50VN (Table S1). PCR reactions were performed using the cDNA with genome-specific forward primers and the anchor reverse primer M10 (Table S1). The 5′ end was recovered with the commercial kit 5′ RACE System (Life Technologies, Carlsbad, CA, USA) according the manufacturer’s instructions using the primers described in Table S1. The last amplified fragments (approximately 350 bp) were gel purified and cloned into pCR™2.1-TOPO^®^ vector (Life Technologies, Carlsbad, CA, USA). Four clones of each fragment were randomly selected and Sanger-sequenced at Macrogen Inc (Seoul, Korea)

### 2.6. Phylogenetic Analysis and Pairwise Comparison

The viral sequences identified were aligned with their corresponding homologs available in GenBank using MAFFT (Multiple Alignment using Fast Fourier Transform) [20]. Phylogenetic trees were inferred using the maximum likelihood method (ML) implemented in FastTree [21], under the generalized time-reversible (GTR) model of nucleotide evolution with gamma-distributed rates among sites (GTR+Γ) or Jones–Taylor–Thornton (JTT) + CAT model of amino acid evolution. Node support was determined using the Shimodaira–Hasegawa (SH) approximate likelihood ratio test (aLRT) [22], which is a nonparametric version of aLRT developed to compare multiple topologies. All trees were midpoint rooted and visualized in FigTree (v1.4.4) [23]. Pairwise identity scores were calculated using SDT version 1.2 [24].

## 3. Results and Discussion

High-throughput sequencing technology and bioinformatics tools have improved the discovery and detection of new viruses. Recently, HTS has been used to describe bean-infecting viruses, such as CPMMV, PvEV-1, PvEV-2, and a newly described Phaseolus vulgaris alphaendornavirus 3 (PvEV-3) [25,26]. Herein, we used HTS to investigate the RNA viruses present in a BGMV-resistant common bean transgenic line with severe symptoms of viral infection (Supplementary Figure S1). The dsRNA extracted (Supplementary Figure S2) from both field and mechanically inoculated samples was pooled and sequenced in MiSeq (Illumina). The raw reads (13,780,310 reads) were processed and assembled using CLC Genomics Workbench, and 27,897 contigs were obtained. Blastx comparisons against a viral RefSeq database revealed six contigs of viral origin ranging in size from 3.4 to 14.8 kb with identities to five different viruses (Table 2). The abundance (i.e., frequency) of each virus also varied from 0.01% to 8.2% of total reads within the pool (Table 2). Four of the identified viruses have already been described infecting common beans and one is a putative new plant rhabdovirus (genus *Cytorhabdovirus*).

The presence of these viruses was confirmed by RT-PCR (Figure S3) and Sanger sequencing. CPMMV, PvEV-1, PvEV-2, and BRMV were detected in the transgenic line CNFCT16207 collected in experimental fields and in the two genotypes of common bean plants mechanically inoculated in the greenhouse (cv. Jalo Precoce and line CNFCT16207). BaCV was amplified only from the plants collected at experimental field and no amplification was observed in the common bean plants mechanically inoculated, suggesting that BaCV is not mechanically transmissible. All virus-derived RT-PCR fragments were confirmed by Sanger sequencing and showed 100% identity with the contigs sequences obtained from the high-throughput sequencing data.

### 3.1. Identification and Genome Assembly of Known Bean-Infecting Viruses

**Figure 1:**
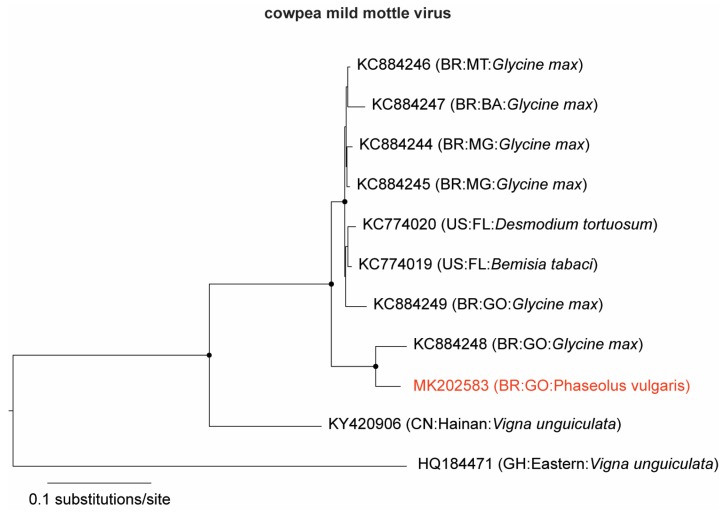
Maximum likelihood tree of cowpea mild mottle virus (CPMMV) isolates. The tree was inferred using an alignment of the complete genomes of CPMMV available in GenBank. The tree is midpoint rooted for purposes of clarity, and the nodes indicated by a black dot have Shimodaira–Hasegawa-like branch test ≥0.9. The newly sequenced CPMMV (BR:GO:14) isolate is highlighted in red.

A contig of 8194 nucleotides (nt) (∼20,000× coverage) was identified as CPMMV (family *Betaflexiviridae*, genus *Carlavirus*) and named CPMMV:BR:GO:14 (Table 2). The contig sequence covered the complete CPMMV genome, with six intact open reading frames (ORFs), the entire untranslated region (UTR), and the 3′ poly (A) tail. In the phylogenetic analysis (Figure 1), CPMMV:BR:GO:14 clustered together with the CPMMV:BR:GO:01:1, isolated in the same region of the country from a soybean plant (*Glycine max*) [27], and in a separate branch from other CPMMV isolates. Next, we carried out a pairwise nucleotide comparison of all complete genomes used in the phylogenetic analysis. When compared with other CPMMV sequences, CPMMV:BR:GO:14 exhibited identity levels ranging from 93% to 96% with six Brazilian CPMMV isolates from soybean [27], with an isolate from *Desmodium tortuosum* and a sequence from whitefly vector-enabled metagenomic (VEM) data from Florida (USA) [28]. CPMMV:BR:GO:14 is the first complete genome sequence of a CPMMV isolate naturally infecting common bean plants.

**Figure 2:**
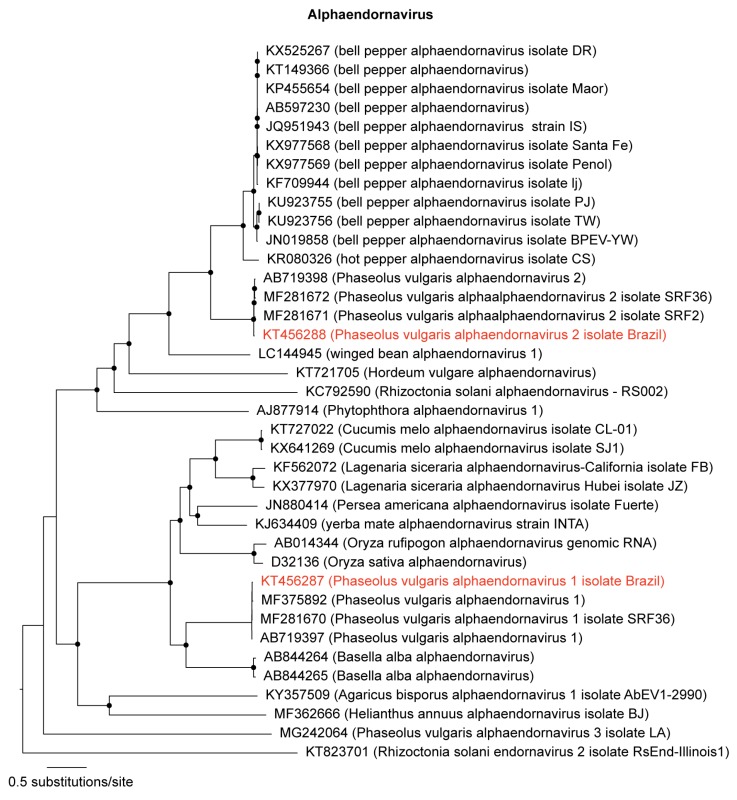
Maximum likelihood tree of alphaendornaviruses. The tree was inferred using an alignment of polyproteins from genomes of all members in the genus *Alphaendornavirus* available in GenBank. The tree is midpoint rooted for purposes of clarity, and the nodes indicated by a black dot have Shimodaira–Hasegawa-like branch test ≥0.9. The newly sequenced alphaendornaviruses are highlighted in red.

Two known members of the *Alphaendornavirus* genus (family *Endornaviridae*) were also identified in our analyses: PvEV-1, and PvEV-2 (Table 2). Their long dsRNA genomes were completely sequenced. The PvEV-1 contig is 14,072 nt long, and the PvEV-2 is 14,817 nt long (Table 2). The sequences were named PvEV-1 [Brazil] and PvEV-2 [Brazil]. Phylogenetic analysis grouped PvEV-1 [Brazil] and PvEV-2 [Brazil] with other members of the *Alphaendornavirus* genus (Figure 2). Sequence analysis showed 99% nt identity among PvEV-1 [Brazil] and PvEV-1 (AB719397) isolated from *P. vulgaris* cultivar Black Turtle Soup from Brazil [3], and 98% with PvEV-1 isolate SRF2 (MF281669) from Kenya. Comparing the PvEV-2 [Brazil] with other isolates, the nucleotide identity score was 97% with PvEV-2 (AB719398) also from Brazil and PvEV-2 isolate SRF2 (MF281671) from Kenya [26].

**Figure 3:**
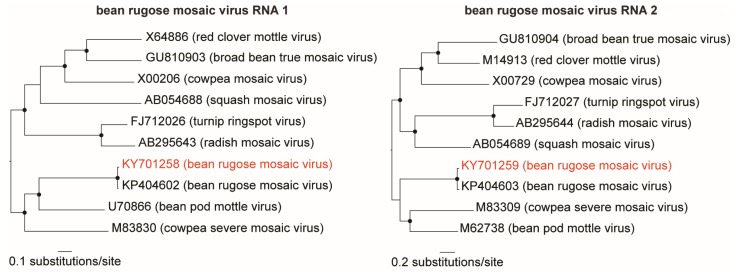
Maximum likelihood trees of comoviruses. The trees were inferred using all available genomes (RNA 1 and RNA 2) of members in the genus *Comovirus*. The trees are midpoint rooted for purposes of clarity, and the nodes indicated by a black dot have Shimodaira–Hasegawa-like branch test ≥0.9. The bean rugose mosaic virus isolate sequenced here is highlighted in red.

Two contigs, sequence of 5891 and 3735 nt (∼3500× and ∼7000× coverage, respectively), were identified as the bipartite bean rugose mosaic virus (BRMV; family *Secoviridae*, genus *Comovirus*) (Table 2). Both genome segments (RNA1 and RNA2) were nearly complete, lacking approximately 13 nucleotides from the UTR ends. Each segment presented an intact ORF encoding a polyprotein of 1856 amino acids (aa) and 1097 aa, respectively. Phylogenetic analysis with other comoviruses grouped BRMV-BR-GO with the BRMV-Paraná isolate (Figure 3). Pairwise sequence analysis showed that the BRMV- BR-GO contigs presented 93% and 92% nt sequence identity with BRMV-Paraná isolate RNA1 (KP404602) and RNA2 (KP404603), respectively, isolated from soybean in southern Brazil [29].

### 3.2. Discovery of a Novel Cytorhabdovirus

**Figure 4:**
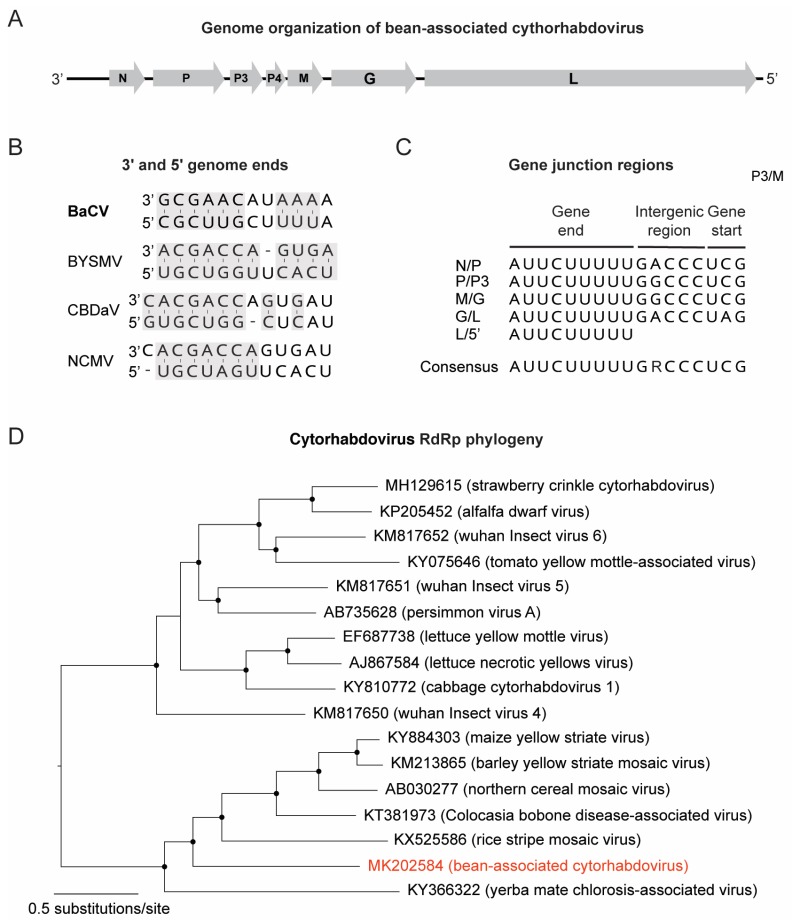
Genome organization and phylogeny of bean-associated cytorhabdovirus (BaCV). (**A**) BaCV genome structure showing the six open reading frames (ORFs) (N, P, P3, M, G, and L). (**B**) Complementary structure of the 5′ and 3′ ends in the BaCV, barley yellow striate mosaic virus (BYSMV), colocasia bobone disease-associated virus (CBDaV), and northern cereal mosaic virus (NCMC) genomes. (**C**) Conserved motif sequences in the intergenic regions. (**D**) Maximum likelihood tree inferred using an alignment of RdRp (L protein) from 17 available genomes of members in the genus *Cytorhabdovirus*. The tree was midpoint rooted for purposes of clarity, and the nodes indicate by a black dot have Shimodaira–Hasegawa-like branch test ≥0.9. BaCV is highlighted in red.

A contig sequence of 13,448 nt (∼26× coverage) showed low similarity (39% aa identity) with the northern cereal mosaic virus (NCMV) RdRP protein (NP_597914.1), a member of the genus *Cytorhabdovirus*. The contig presented the typical genomic organization of the viruses members of the family *Rhabdoviridae*, including a 3′ leader followed by seven open reading frames (ORFs) and a 5′ trailer sequence [30] (Figure 4A), and is tentatively named bean-associated cytorhabdovirus (BaCV) (Table 2). The 3′ and 5′ ends were obtained through RACE and the complete genome size is 13,467 nt long. As shown in Figure 4B, the terminal nucleotides (∼12 nt) from the leader (145 nt) and the trailer (58 nt) regions are partially complementary to each other (highlighted in grey), similarly to what is observed for other cytorhabdoviruses like barley yellow striate mosaic virus (BYSMV), colocasia bobone disease-associated virus (CBDaV), and NCMV. Each ORF is separated by a conserved gene junction (consensus 3′-AUUCUUUUUGRCCCUMG-5′), except for the 3′/N region and P3/P4 (Figure 4C). The putative 3′ poly-adenylation signal (the 3′ end of the mRNA) was completely conserved in all junctions (3’-AUUCUUUUU-5′), while the intergenic spacer (non-transcribed sequence) and the putative transcription initiation sequence (5′ end of each subsequent mRNA) presented minor variations (Figure 4C). The putative transcription initiation sequence for N mRNA was confirmed by RACE at the genomic sequence 3’-AUUCU-5’ (16 nt upstream the initiation codon).

The viral genome presented all five ORFs found in most rhabdoviruses: nucleoprotein (N) (ORF1) (451 aa), phosphoprotein (P) (ORF2) (445 aa), matrix (M) (ORF5) (287 aa), glycoprotein (G) (ORF6) (520 aa), and an RNA-dependent RNA polymerase (L) (ORF7) (2114 aa), as well as a putative movement protein (P3) (ORF3) (189 aa) and a hypothetical protein P4 (ORF4, 78 aa) (Table 3), which is also present in the genome of other members of the genus *Cytorhabdovirus* [30]. The predicted ORFs 3 and 4 are not separated by the conserved gene junction described above. A putative homolog of this small protein (P4) was recently described in the genome of yerba mate chlorosis-associated virus (YmCaV) (P4 protein, ARA91089), which also does not have any conserved gene junction between P3 and P4 genes [31]. Consecutive ORFs, expressed from the same mRNA and separated by a short stretch of nucleotides, are common in several rhabdoviruses and depend on a conserved element upstream of the restart AUG called the termination upstream ribosome binding site (TURBS) [32]. No TURBS motif was found upstream the potential start codon of this predict small ORF of BaCV. Therefore, the bicistronic nature of ORF3 mRNA remains to be experimentally confirmed in the future.

Moreover, the predicted BaCV proteins were compared to homologous proteins from the closest cytorhabdoviruses, revealing a low level of sequence identity, ranging from 15% to 39% (Table 4). The nucleoprotein and RdRp were the most conserved proteins (Table 4), while P3 and M were the most divergent. Overall, the closest related virus varied according to the viral protein, suggesting that such low level of sequence identity may indeed misrepresent the actual distances between these viruses. Therefore, to investigate the evolutionary relationships of BaCV and other cytorhabdoviruses, a maximum likelihood phylogeny was inferred based on a multiple alignment of RdRp. The phylogenetic analysis shows that cytorhabdoviruses can be divided into two highly supported clades, one comprising viruses that predominantly infect dicots, like alfalfa dwarf virus (ADV) and lettuce yellow mottle virus (LYMoV), and the other containing viruses that infect monocots and dicots (Figure 4D), like NCMV, MYSV, CBDaV, and YmCaV. BaCV clustered within the latter and was mostly related to YmCaV and RSMV (Figure 4D). Crucially, the genome organization of BaCV, YmCaV and RSMV is similar, except that RSMV and YmCaV encode an additional protein in their genomes, P6 and P4 proteins, respectively. Overall, these results provide strong evidence that BaCV is indeed a new virus species in the genus *Cytorhabdovirus* (family *Rhabdoviridae*).

In summary, HTS was instrumental in uncovering RNA viruses infecting BGMV-resistant transgenic plants presenting severe symptoms in the field. Our findings extend the knowledge of bean-infecting viruses in Brazil and warrants further studies on the biology, insect transmission, and distribution of BaCV, the first cytorhabdovirus to be identified infecting common beans.

## Figures and Tables

**Table 1:** Virus-specific primers sequences used for RT-PCR.

Genus	Primer Name	Sequence 5′-3′	Amplicon (bp)	Tm (°C)
*Alphaendornavirus*	PvEV-1 ^1^ F	GTAAACCAGGGAATTGGTGG ^5^	303	60
	PvEV-1 ^1^ R	GATTGATTGGGCTGTATAGTG ^5^		
	PvEV-2 ^1^ F	TGTTAGGCGTGTGTCCCCA ^5^	519	56
	PvEv-2 ^1^ R	GTTGCTGTATTGCTCGTGTC ^5^		
*Comovirus*	BRMV ^2^-RNA1-2550 F	GACAATACAGCCTATGATGGGA	995	61
	BRMV ^2^-RNA1-3545 R	CACCAATGATCCACAATCCA		
	BRMV ^2^-RNA2-1704 F	TCTGGTGATGGGTTATTTTCTCAGA	1832	60
	BRMV ^2^-RNA2-3536 R	CTACTGATACATCCTATCCATTGCA		
*Carlavirus*	CPMMV ^3^-4000 F	AACTTGGCCTTAGTGAACTCTACA	500	58
	CPMMV ^3^-4500 R	ATTAGCTCTGTGCCTGGGGT		
*Cytorhabdovirus*	BaCV ^4^-6491 F	GAAGTCGCATAGCTCGTCGA	687	60
	BaCV ^4^-7178 R	GAGCGATAAGAACCTCCCCG		

^1^ Phaseolus vulgaris alphaendornavirus 1 and 2, ^2^ bean rugose mosaic virus and, ^3^ cowpea mild mottle virus, ^4^ bean-associated cytorhabdovirus. ^5^ [3].

**Table 2:** Viral genomes found infecting the bean golden mosaic virus (BGMV)-resistant CNFCT16207 common bean transgenic line.

GenBank Accession Number	Length (nt)	Mapped Reads	% of Total Reads	Coverage	Classification		
					Genus	Virus	Isolate
MK202583	8194	1,131,964	8.2	19,810×	*Carlavirus*	CPMMV ^1^	CPMMV:BR:GO:14
KT456287	14,072	73,996	0.5	762×	*Alphaendornavirus*	PvEV-1 ^2^	PvEV-1 (Brazil)
KT456288	14,817	114,256	0.8	1123×	*Alphaendornavirus*	PvEV-2 ^2^	PvEV-2 (Brazil)
KY701258	5891	151,744	1.1	3500×	*Comovirus*	BRMV-RNA1 ^3^	BRMV- BR-GO
KY701259	3735	182,331	1.3	7000×		BRMV-RNA2 ^3^	BRMV- BR-GO
MK202584	13,467	2400	0.01	26×	*Cytorhabdovirus*	BaCV ^4^	BacV-BR-GO

^1^ cowpea mild mottle virus, ^2^ Phaseolus vulgaris alphaendornavirus 1 and 2, ^3^ bean rugose mosaic virus and ^4^ bean-associated cytorhabdovirus.

**Table 3:** Features of the ORFs encoded by bean-associated cytorhabdovirus.

ORFs ^1^	Size (nt/aa)	Putative Product	Blastp Hit (Organism) (Acession)
1	1356/451	Nucleoprotein (N)	Nucleocapsid protein (RSMV ^3^) (APR74648)
2	1338/445	Phosphoprotein (P)	Phosphoprotein (BYSMV ^4^) (AJW82843)
3	570/189	Movement protein (P3)	Gene 3 protein (NCMV ^5^) (NP_057956)
4	237/79	Hypothetical protein (P4)	No hit
5	645/287	Matrix protein (M)	No hit
6	1560/520	Glycoprotein (G)	Glycoprotein (YmCaV ^6^) (ARA91090)
7	6342/2114	RdRP ^2^ (L)	RdRp ^2^ (BYSMV ^4^) (YP_009177231)

^1^ Open reading frame; ^2^ RNA-dependent RNA polymerase; ^3^ rice stripe mosaic virus; ^4^ barley yellow striate mosaic virus; ^5^ northern cereal mosaic virus; ^6^ yerba mate chlorosis-associated virus.

**Table 4:** Amino acid sequence identities (%) of bean-associated cytorhabdovirus (BaCV) proteins compared with their homologs of other cytorhabdoviruses.

GenBank Accession	Virus	N	P	P3	M	G	L
KY366322	YmCaV ^1^	27	20	**25**	**21**	23	33
KX525586	RSMV ^2^	**30**	24	23	17	22	35
KT381973	CBDaV ^3^	26	24	19	18	21	38
AB030277	NCMV ^4^	26	23	23	16	24	**39**
KM213865	BYSM ^5^	26	**26**	21	18	**25**	**39**
KY884303	MYSV ^6^	29	22	19	15	23	**39**

^1^ yerba mate chlorosis-associated virus; ^2^ rice stripe mosaic virus; ^3^ colocasia bobone disease-associated virus; ^4^ northern cereal mosaic virus; ^5^ barley yellow striate mosaic virus; ^6^ maize yellow striate virus. Highest percent identities are in bold.

## Supplementary Materials

The following are available online at http://www.mdpi.com/1999-4915/11/1/90/s1, Figure S1. Symptoms displayed by bean transgenic line CNFCT16207 in the field (A) and mechanically inoculated plants in the greenhouse (B). Figure S2. Agarose gel electrophoresis of dsRNA extracted from: (1) Bean transgenic line CNFCT16207 collected in experimental fields; (2) mechanically inoculated common bean cv. Jalo Precoce and (M) 1 kb Plus DNA Ladder. Figure S3: RT-PCR detection of PvEV-1, PvEv-2, CPMMV, BRMV RNA-1 and RNA2, and BaCV using specific primers: Plant line CNFCT16207 collected in an experimental field (1 and 2), bean plants mechanically inoculated in the greenhouse cv. Jalo Precoce (3) and line CNFCT16207 (4); negative control (C-); 1 kb Plus DNA Ladder. Table S1: Primers sequences used for RACE.
