# Supplementary material for: Double-Stranded RNA High-Throughput Sequencing Reveals a New Cytorhabdovirus in a Bean Golden Mosaic Virus-Resistant Common Bean Transgenic Line
Source: Viruses. 2019 Jan 21;11(1):90. doi: 10.3390/v11010090 (PMC6357046; doi:10.3390/v11010090)
Supplement: Supplementary file 1 [file viruses-11-00090-s001.pdf]

Supplementary material

Supplementary Table

Table S1. Primers sequences used for RACE.

RACE	Primer name[1]	Primer sequence 5'-3'	Amplicon size (bp)	Tm (°C)
5' First PCR	AAP	GGCCACGCGTCGACTAGTACGGGIIGGGIIGGGIIG	600	61
	BaCV-546 R	TTCACTCTCCTCAGACTCCTTGC		
5' Second PCR	AUAP	GGCCACGCGTCGACTAGTAC	350	61
	BaCV-321 R	TGTACATCCCATACCGCTCCAG		
3' First PCR	M10[2]	AAGCAGTGTTATCAACGCAGA	600	61
	BaCV-13054 F	TGGGACTGAAGACGACAACG		
3' Second PCR	M10[2]	AAGCAGTGTTATCAACGCAGA	350	61
	BaCV-13169 F	GGTGACCTTCCAGTACCTTCCTC		
3' - cDNA	M10PacIT50VN[2]	AAGCAGTGTTATCAACGCAGATTAATTAAT50VN	-	-

[1] Primer F- forward sense; R- reverse sense. [2] Nicolini et al. 2012.

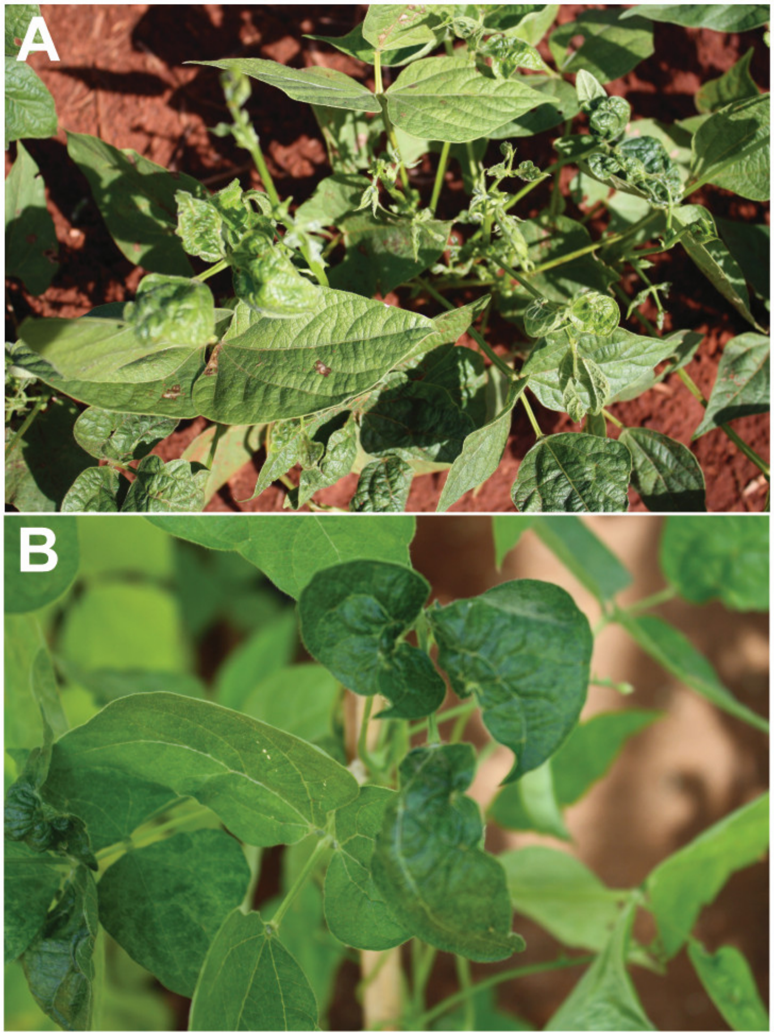

Supplementary Figure 1. Symptoms displayed by bean transgenic line CNFCT16207 in the field (A) and mechanically inoculated plant in the greenhouse (B).

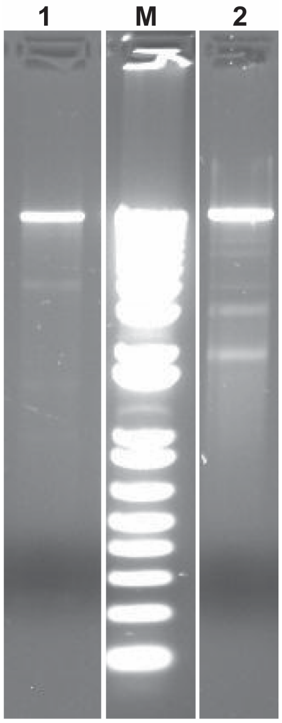

Supplementary Figure 2. Agarose gel electrophoresis of dsRNA extracted from: (1) Bean transgenic line CNFCT16207 collected in experimental fields; (2) mechanically inoculated common bean cv Jalo Precoce and (M) 1 kb Plus DNA Ladder.

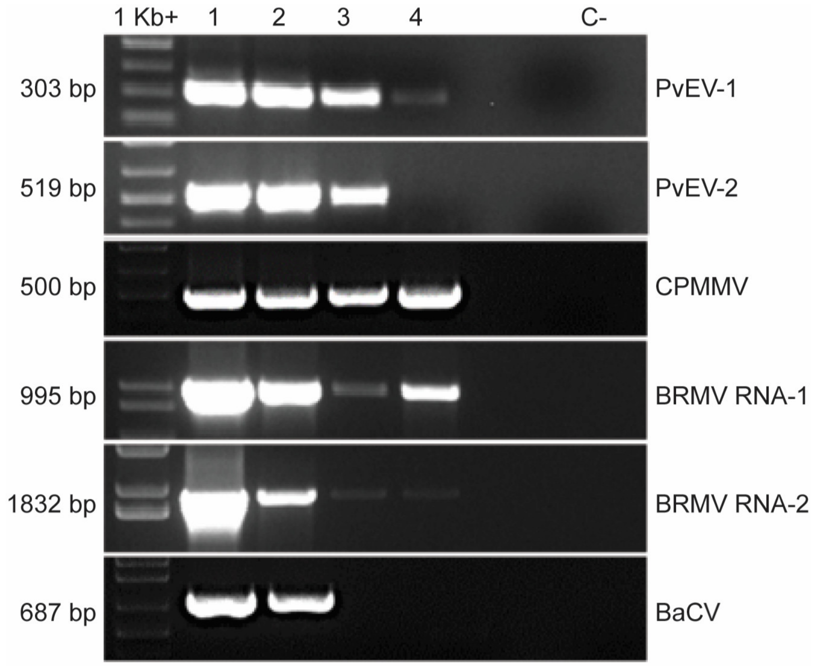

Supplementary Figure 3. RT-PCR detection of PvEV-1, PvEv-2, CPMMV, BRMV RNA-1 and RNA-2, and BaCV using specific primers. Agarose gel electrophoresis of virus-derived amplicons from bean plants of line CNFCT16207 collected in an experimental field (**1** and **2**), bean plants mechanically inoculated in the greenhouse, cv Jalo Precoce (**3**) transgenic line CNFCT16207 (**4**); no template control (**C-**); 1 kb Plus DNA Ladder.
